# A Novel Compact Multi-Reflecting Time-of-Flight Mass Spectrometer

**DOI:** 10.1021/jasms.5c00321

**Published:** 2026-01-26

**Authors:** Anatoly N. Verenchikov, Jason Wildgoose, Sergey N. Kirillov, Aleksey V. Vorobyev, Vasily V. Makarov, Lee A. Gethings, Robert P. Tonge, Matthew E. Daly, William J. Johnson, James I. Langridge

**Affiliations:** † Mass Spectrometry Consulting Ltd, A5 JNA blv, Bar 85000, Montenegro; ‡ 36565 Waters Corporation, Altrincham Road, Wilmslow SK9 4AX, United Kingdom

**Keywords:** multireflecting time-of-flight, high resolution, lipidomics

## Abstract

Mass spectrometry is an indispensable tool for the rapid and in-depth analysis of complex mixtures across diverse biologically important fields including metabolomics, lipidomics, and proteomics. These applications demand high speed instruments with subppm mass measurement accuracy over a wide dynamic range of sample concentrations. Here, we introduce an liquid chromatography–mass spectrometry/MS (LC-MS/MS) quadrupole time-of-flight mass spectrometer featuring a novel collision cell, a high dynamic range detector, and a compact multireflecting orthogonal time-of-flight analyzer. This innovative instrument achieves high analytical performance, acquiring full mass range spectra at 100,000 Full Width Half Maximum (FWHM) resolution up to 100 spectra/s acquisition speed. The instrument achieves excellent linearity within a dynamic range of 10^5^, with a correlation coefficient *R* ^2^ = 0.984. The speed, resolution and dynamic range are in excellent balance as demonstrated by the analysis of isotopically labeled lipids in human blood plasma.

## Graphical abstract

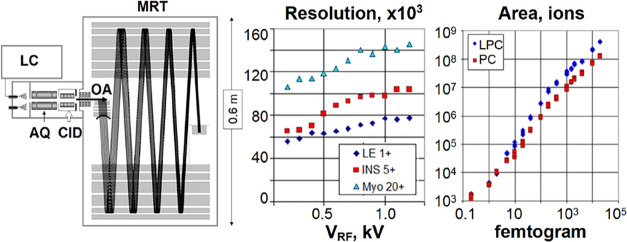

## Introduction

Time-of-Flight Mass Spectrometry (TOFMS) has been a cornerstone analytical technology, experiencing several periods of resurgence within mass spectrometry.^1^ The evolution began with GC-TOF in the 1960s, followed by a renaissance in the 1990s driven by the introduction of Matrix-Assisted Laser Desorption/Ionization (MALDI)^2–5^ and the rapid MS/MS capabilities of ESI-Q-TOF instruments.^6,7^ Orbitrap analyzer based mass spectrometers were introduced in the 2000s,^8,9^ which also excelled in resolution, mass accuracy, and sensitivity, significantly impacting the rapidly evolving field of proteomics.

However, as demands for higher throughput and improved ion source sensitivity increased, limitations in speed and dynamic range became evident. Modern ESI sources can generate ion fluxes reaching 6 × 10^9^ ions/s. This significantly exceeds the capacity of Orbitrap analyzers, which can typically handle not more than 10^6^ charges/spectrum at low analysis frequencies (2 Hz), while maintaining 100,000 FWHM resolution.^9^ The effective utilization of the ion influx is limited compared to the claimed 90% duty cycle of ion trapping pulse converters.^9^ Furthermore, the coalescence effect severely restricts the intensity of individual peaks when resolving closely spaced isobars,^10^ obscuring the robust and high-throughput analysis of complex mixtures with overlapping isotopic patterns.^11^

A TOFMS resurgence was driven by the development of multireflecting instruments (MRTs).^12,13^ MRT technology offers high resolution comparable to Orbitraps, but at significantly higher analysis speeds. Commercial examples include the GC-HRT,^14^ SELECT SERIES MRT,^15^ Astral,^16^ and the Xevo MRT mass spectrometer presented here. The difference in ion optical schemes and parameters of these MRT analyzers is thoroughly described in the recent review.^13^

Early MRT instruments focused on improving resolution through substantial elongation of the folded flight path, particularly within the *Y*-injection scheme.^13^ In Y-MRT, a continuous ion beam is injected orthogonal to the analyzer’s midplane (along the *Y*-axis). This enables narrow beam width (1–2 mm) in the drift *Z*-direction, a dense folding (at 10 mm *Z*-pitch) and substantial elongation of ion trajectory in the analyzer. Despite long flight path (100 m), ion packets stay confined with the aid of a periodic lens in the drift space. While achieving resolution up to 1,000,000,^17^ Y-MRT designs suffered from low duty cycles affected by both short ion packets *Y*-length (5 mm) and long period (2.5 ms for *m*/*z* = 1000 Th). Duty cycle enhancements via encoded frequent pulsing^12^ and the Pulsar method^18^ improved throughput and allowed to recover the duty cycle up to 10% but encountered dynamic range limitations at the spectral decoding step or space charge limitations in the Pulsar method.

Over the past decade, researchers have explored an alternative MRT design utilizing a conventional orthogonal accelerator (OA) aligned with the drift direction, analogous to OA-TOFMS.^19–21^ This orthogonal-MRT (o-MRT) scheme allows for an extended OA, significantly boosting the duty cycle, further extended with ion accumulation in radiofrequency ion guides.^22,23^ Additionally, aligning ion packets along the drift axis minimizes analyzer aberrations, further enhanced by optimized ion mirror fields. To distinguish this design from Y-injection MRT, we refer to it as “o-MRT.” Several o-MRT prototypes have been developed using both ElectroSpray Ionization (ESI)^24^ and electron ionization (EI)^25^ ion sources.

The o-MRT concept has been further developed and recently commercialized by Waters Corporation with the introduction of the Xevo-MRT at the Annual Conference on Mass Spectrometry and Allied Topics in 2024. Here, we report details of the instrument design, including ion simulation data, and some of the key performance measures resolution, dynamic range, sensitivity, and mass accuracy. We then illustrate the application of the instrument to an liquid chromatography–mass spectrometry (LC-MS) lipidomics workflow with human blood plasma lipids.

Lipid identification and quantification in biological samples poses significant analytical challenges.^26^ The inherent complexity of biological samples, including the wide dynamic range and matrix effects, leads to frequent chromatographic and mass separation overlaps. Furthermore, instrument sensitivity can vary by orders of magnitude for specific analyte types and classes. These factors necessitate mass spectrometers with high dynamic range (>10^4^), resolution (>100,000 FWHM), and mass measurement accuracy (<1 ppm RMS) that allow for reliable lipid analysis.

## Experimental Section

### Instrument Design and Operation

#### Design Overview of the Quadrupole Compact MRT Instrument

The compact-MRT design is shown in Figure 1A, consisting of the following components: 1- LC separation systems; 2 - closed ion source, accepting various ionization modes and probe types; 3 - a heated nozzle with gas channels; 4 - dual stage traveling wave ion guides (TWIG); 5- MS1- analytical quadrupole for precursor ion selection; 6 - collision cell; 7 - lens system; 8- gridless orthogonal accelerator (OA) with a 9 -*trans*-axial exit lens; 10 - MRT housing; 11 - opposed gridless ion mirrors; 12 - simulated ion trajectories; 13- floated drift space; 14 - detector; 15 - dual gain preamplifier; 16 - ADC; 17 - GPU for data processing and PCU for data summation and storage. The white arrows show the placement of the mechanical and turbo-molecular pumps. Each subcomponent can be described in more detail as follows.

**Figure 1:**
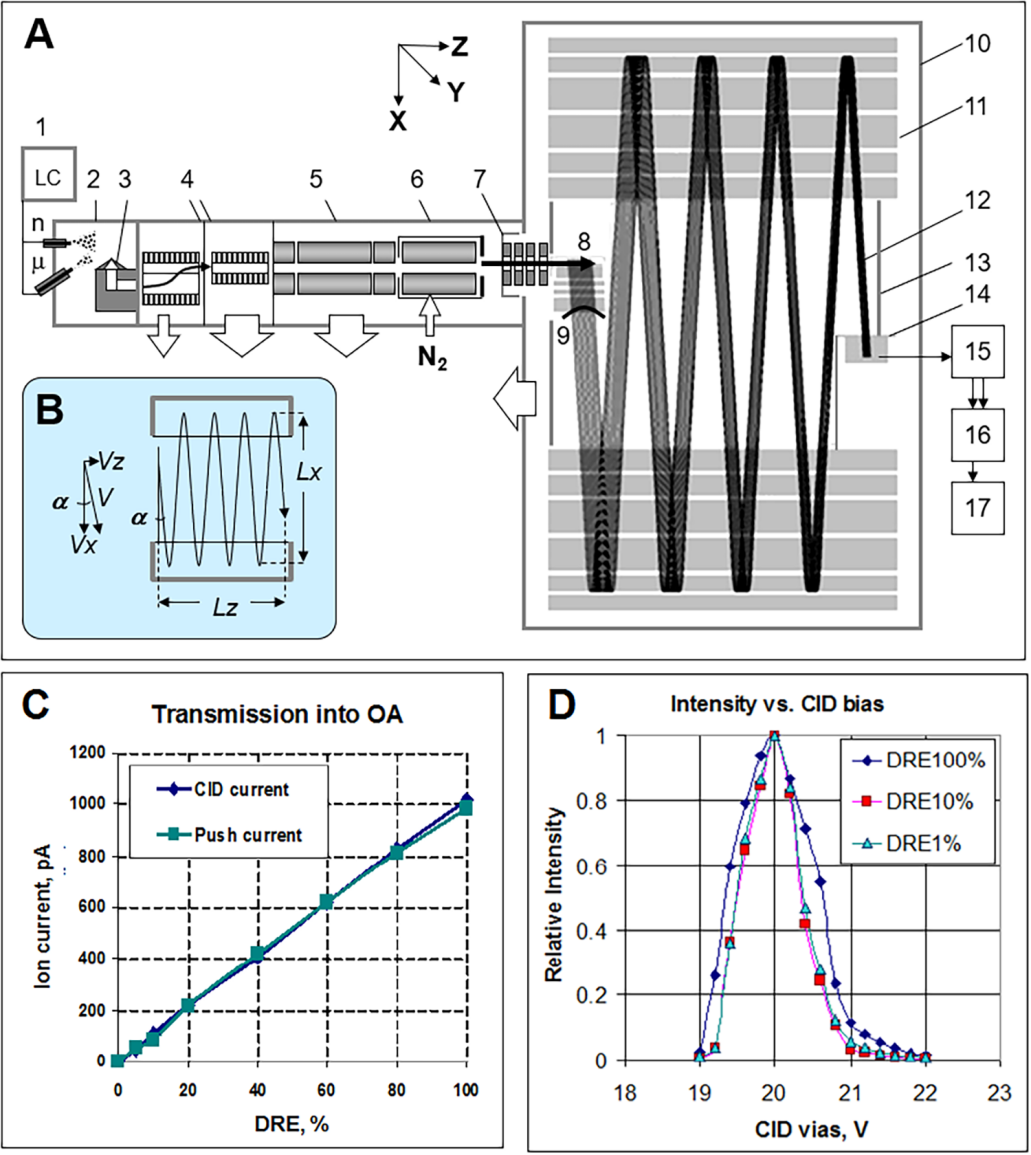
Overview of Xevo MRT: (A) Main components of the instrument and simulated ion trajectories in the analyzer. (B) Principles of the ion trajectory control and the main analyzer dimensions (500 mm (Lx) and 250 mm (Lz)). (C) Ion currents into the gas cell and accelerator (measured on the Push electrode) vs interface muting with the method of Dynamic Range Enhancement (DRE). (D) Ion transmission onto the detector vs gas cell bias, illustrating the control of ion trajectories and effect of stronger ion currents in the gas cell.

#### LC and ESI Source (Figure 1A, Components **1–2**)

An ACQUITY Premier FTN UPLC system (Waters Corporation, Milford, MA), incorporating a flow-through needle and ACQUITY Premier CSH C18 Column 1.7 μm, 2.1 mm × 100 mm (Waters Corporation, Milford, MA) separation column was coupled to the closed ESI source for the applications described in this manuscript. Besides conventional ESI, nanoflow ESI or Atmospheric Pressure Chemical Ionization (APCI) sources can be interfaced to the instrument. All probes utilize a nebulizing gas for spray stability. An additional ESI probe is fed with a reference compound with the aid of a Waters Reagent Delivery System (Waters Corporation, Wilmslow, U.K.) or a syringe pump, which can be alternately selected using an electrically driven baffle

#### Front-End Interface (Figure 1A, Components **3–4**)

Ions are sampled via heated cone with a 0.5 mm aperture (Nozzle, 1) followed by a curved heated channel for the removal of the ESI aerosol. Two stages of Traveling Wave Ion Guide (TW, 2) are pumped by a 100 m^3^/h dry pump (EV-SA20 Ebara, Japan) and the first stage of a dual stage turbo-molecular pump operating at ∼200 L/s (Edwards, U.K.) to gas pressures of ∼2.5 mbar and ∼2 × 10^–2^ mbar, respectively. The first TW-guide has two offset channels to remove droplet residuals. The combination of Radio Frequency (RF) and TW signals in ion guides allow for radial ion confinement and for rapid ion transfer, which eliminates space charge build up. The front-end interface is characterized by an effective removal of ESI droplets and by high ion transmission range ion currents.

#### Analytical Quadrupole (AQ) (Figure 1A, Component **5**)

A 130 mm long AQ (5) with 10 mm inscribed diameter is used for precursor ion selection in MS/MS experiments. Entrance and exit Brubaker rods of 15 mm length are used to enhance the AQ ion transmission close to 100% when selecting mass windows greater than 2 *m*/*z*. The AQ is terminated by a timed gate for rapid and quantitative suppression of ion signal, providing Dynamic Range Enhancement (DRE) functionality as detailed below.^27^ The AQ region is pumped down by the second stage of a dual stage turbo-molecular pump operating at ∼300 L/s (Edwards, U.K.) to a gas pressure of ∼3 × 10^–5^ mbar. The AQ is energized by an RF/DC generator. For a mass range up to 8000 *m*/*z*, the RF frequency is 597 kHz. The generator allows for rapid mass scanning (up to 10,000 Da/s), fast switching (down to 1 ms dwell time), and for adjustments of mass selection window (from 2 to 100 *m*/*z*). Furthermore, the generator allows to transmit a wide mass range by switching off the DC voltage between rods, affording rapid switching between full mass range and selected mass range transmissions.

#### Gas Cell (Figure 1A, Component **6**)

The gas cell is a key component of the instrument, specifically designed for efficient ion manipulation. This sealed cell operates with N_2_ gas (indicated by a white arrow in Figure 1) at a pressure of ∼1.5 × 10^–2^ mbar. The gas cell entrance features a 2 mm diameter aperture that is designed to efficiently capture ions over a wide range of energy to charge ratios (2–100 V) from the AQ. The 3 mm diameter segmented quadrupole provides enhanced ion confinement enabling high transmission to and through downstream devices. The cell is powered by an RF generator operating at a frequency of 5 MHz and a programmable amplitude of up to 1.2 kV (peak-to-peak). This configuration allows for transmission of a broad mass range of ions, starting from as low as *m*/*z* 50.

The collision energy (CE), which refers to the ion energy upon injection into the gas cell, is controlled by a DC bias applied to the front-end interface. This allows for rapid switching of the injection energy within 1 ms. At low CE values (3–5 V), ions primarily pass through the cell with minimal fragmentation. In MS/MS experiments, the CE is dynamically adjusted based on the mass-to-charge ratio (*m*/*z*) and charge (*z*) of the selected ions to achieve optimal fragmentation for structural elucidation.

Axial DC voltage gradients between segments within the gas cell enable rapid MS/MS adjustments. This design allows swift ion transfer within the cell, typically on a 1 ms time scale. The energy of ions exiting the cell is controlled by the DC bias applied to the exit portion. To maintain a constant ion beam energy, the exit DC bias is typically set to *Kz* = 22 V. A combination of axial DC gradients and of sufficient ion damping results in a low spread in the axial ion energy-to-charge ratio (*dKz*) of typically less than 0.5 V, even when transmitting ion currents up to 1 nA. As described below, the low energy spread is essential for effective ion transmission.

#### Ion Optics (Figure 1A, Component **7**)

Following the collision cell, a sophisticated ion optics system refocuses the ion beam, shaping it into a near-parallel configuration with a magnified diameter (×5) and significantly reduced angular divergence. The resulting beam exhibits a mean ion energy-to-charge ratio (*Kz*) of 22 V, low axial energy spread (*dKz* ∼ 0.5 V), and beam diameter within 1 mm and at typical FWHM of 0.6–0.7 mm. These characteristics can vary slightly based on the initial compression in the gas cell (mass and charge dependent).

The ion optics utilizes a telescopic set of Einsel lens for an intermediate crossover, improving tolerance to misalignments. Two sets of deflectors ensure precise beam alignment before entering the orthogonal accelerator (OA). Finally, a heated collimator (1 mm diameter) removes unwanted beam wings and maintains high transmission efficiency (>90%) for currents up to 1 nA, as illustrated by experimental measurements presented in Figure 1C. In these experiments, a 10 μM solution of insulin was infused, introducing approximately 1 nA ion current into the gas cell. The current was estimated by timed gates past AQ, following the method of DRE. In the wide range of deeming DRE factor, the directly measured ion current entering the OA, appears nearly equal to one entering RFG, this way proving efficient ion transfer by the lens system, occurring in a wide range of ion currents, up to 1 nA.

#### Orthogonal Accelerator (Figure 1A, Components **8–9**)

The OA transforms the continuous ion beam into tightly focused packets with minimal energy spread and with spatial ion focusing, essential conditions for instrument performance. Aligned with the instrument’s drift axis *Z*, the gridless OA utilizes parallel plates with narrow slits (5 mm) for efficient ion transmission. Thick plates (minimum 2 mm) ensure mechanical stability. To minimize electrical leakage (below 300 V/mm on insulator surfaces), high-voltage electrodes utilize stepped designs with thick insulating spacers. The core function of the OA is to create well-defined ion packets for injection into the analyzer.

An 8 mm “pusher gap” acts in conjunction with combined push–pull voltage totaling 3050 V generating a strong electric field of 380 V/mm. This configuration leads to a low turnaround time (down to ∼0.2 ns) at a moderate energy spread of around 260 V, corresponding to less than 5% of the mean ion energy per charge (∼6600 V). Both help to maintain good mass resolution in the subsequent analyzer stage. A second pull electrode minimizes the penetration of the constant accelerating field during the ion filling stage.

The elongated design allows for the extraction of longer ion packets (up to 22 mm), effectively enhancing the overall duty cycle of the instrument. Careful design minimizes fringing fields in the *Z*-direction to prevent unwanted ion steering during packet transport. Reduced electrode rims and offset placement relative to the analyzer’s central plane ensure minimal disruption to ion packets after their first mirror reflection. Focusing elements within the OA include: *Y*-direction Lens, also featuring split electrodes to allow for minor beam steering, and a *Z*-direction trans-axial lens. The intentional curvature of the pusher gap’s electric field acts to correct for second-order aberrations within the lenses.^28^

#### MRT Analyzer (Figure 1A, Component **10** and Internals)

The design of the analyzer offers exceptional focusing capabilities and a high-vacuum environment, making it well-suited for high-resolution analysis for a variety of biomolecules. A planar design is employed, consisting of two parallel ion mirrors with rectangular frames elongated in the *Z*-direction (drift direction) for enhanced focusing. Thick electrodes ensure mechanical stability and precise mirror alignment (∼20 μm).

The analyzer prioritizes grid-free designs for both the OA and ion mirrors. This approach eliminates potential ion losses that could occur due to interactions with meshes during multiple ion reflections. For instance, if an MRT instrument with *N* = 8 reflections was constructed using grid-covered dual-stage mirrors, the overall analyzer transmission would plummet to a mere 3% (0.9^32^) - assuming a 90% transmission efficiency per individual mesh. Key features of the analyzer include:

##### Vacuum System

An electroless nickel-plated aluminum housing with dual O-ring seals maintains a high vacuum level (∼1 × 10^–7^ mbar.) using a single turbo-molecular pump (300 L/s, Pfeiffer, Germany). This configuration is well-suited for analyzing a wide range of analyte molecular sizes due to the negligible ion-gas scattering at this pressure for lighter ions.

##### Full Third-Order Focusing

The ion mirror design achieves exceptional focusing, minimizing aberrations and enabling high resolution (*R* = 100,000) for typical ion packet characteristics (less than 5% energy spread and less than 10% packet width compared to the mirror window). Details are in following ion optical section.

##### *Y*-Direction Focusing

The *Z*-orientation of ion packets naturally helps maintain a narrow profile in the *Y*-direction (typically within 1–2 mm). Additionally, minor Y-steering within the OA allows for precise alignment of the packets with the central plane of the mirrors. Periodic confinement by the mirrors further contributes to this *Y*-direction focusing and improves packet localization, ultimately reducing the time spread at the detector.

##### *Z*-Direction Trajectory (Figure 1B)

The mirror length and positioning are optimized to provide a usable ion trajectory length of at least 250 mm between the OA and the detector (14) while accommodating 22 mm long ion packets. Mutual shifting of the mirrors maximizes the distance between ions and reduces effects of fringing fields in the *Z*-direction. Notably, the analyzer relies primarily on controlled ion beam energy and spatial focusing past the OA for *Z*-direction motion. Ion packets freely propagate in this direction.

#### Detector (Figure 1A, Component **15**)

The instrument presents challenges for detector selection due to the combination of a relatively short ion packets (down to 0.2 ns) and high ion fluxes (up to 1 × 10^9^ ion/s). A commercially available, fast electron multiplier, employing magnetic focusing optics was selected. This detector offers an exceptional combination of performance characteristics including, low single ion time spread (∼500 ps FWHM), wide dynamic range (∼1 mV to ∼2.5 V output pulse linearity), large detection window (35 mm × 15 mm) and exceptional surface flatness (±10 μm).

#### Data System (Figure 1A, Components **16–17**)

To explain the dynamic range requirements, lambda (λ) is introduced. Lambda represents the number of ions per peak per orthogonal acceleration push into the MRT analyzer, corresponding to the number of ions within a single ion packet. Even in complex mixtures, some components may have high concentrations, leading to peak signals reaching λ = 5000, translating to 25 × 10^6^ ions/peak/s at a pulsing rate of 5 kHz. Even higher lambdas are possible for simpler and more concentrated samples.

A single-channel data acquisition system is limited in dynamic range due to the need to detect single ions with a signal-to-noise ratio (SNR) greater than 10 to provide for efficient ion detection at a wide amplitude spread per single ion. Additionally, both fast preamplifiers and fast analog-to-digital converters (ADCs) have limitations of signal-to-noise ratio SNR < 1000. The Compact-MRT instrument overcomes this limitation by employing a dual-channel data system with a selected gain ratio of 64 featuring an SA240P ADC card with two parallel channels operating at 4 GS/s (0.25 ns per point) and 14-bit vertical resolution. The detector signal is split between two parallel amplifier channels and recorded simultaneously in both ADC channels at 9 bits per channel before transmission via the ADC bus. This allows for real-time signal processing on a graphics processing unit (GPU) leveraging its parallel processing capabilities.

#### Data Acquisition

The instrument employs real time data centroiding which minimizes the effects of detector time spread that can broaden perceived peak widths and limit resolution. The centroid method leverages the Xevo MRT’s data system for precise information from individual TOF spectra every 140–260 μs, which involves ADC sampling of the TOF spectra (4 GHz), compression of the TOF data (<5 GB/s), transfer of data to the GPU for ion peak identification, centering, and area calculation. The centroid times are represented on a finer time grid than those offered by the initial 4 GHz sampling rate. This approach significantly reduces time spreading due to the detector response (0.6 to 0.2 ns)­and reflects the true arrival time distribution of ions measured separated and measured by MRT analyzer.

#### Ion Injection

Here we describe the efficiency of pulsed conversion in the OA and the principles behind ion trajectory control within the *Z*–*X* plane of the instrument.

##### Orthogonal Acceleration (Figure 1A)

A continuous or time-modulated ion beam exits the gas cell as shown by a wide black arrow. The lens system refocuses the beam before it enters the OA along the *Z*-axis, balancing a minimal angular divergence with the tolerable beam diameter (under 1 mm). The DC bias of the gas cell controls the average ion energy per charge (*Kz*) inside the OA’s pushing gap. Periodic extraction pulses accelerate ion packets in the *X*-direction (orthogonal to Z) to an energy per charge (*Kx*) defined by an acceleration voltage (*U*_acc_) and the amplitude of the pulses (push and pull).

##### Trajectory Inclination (Figure 1B)

Extracted ion packets retain the initial z-component of continuous ion beam energy. This results in ion packets traveling at a small inclination angle (α) relative to the time-of-flight *X*-axis, following a zigzag trajectory (12). Due to the absence of additional control mechanisms within the analyzer, the trajectory inclination remains constant throughout the drift space. Equations 1 and 2 below describe these processes and highlight the decoupled nature of ion motion in the *X* and *Z* directions

1 $$\mathrm{Kx}=U_{\mathrm{acc}}+\left(\mathrm{push}+\mathrm{pull}\right)/2$$

2 $$a=\mathrm{arctg}\left(\mathrm{Vz}/\mathrm{Vx}\right)∼\mathrm{Vz}/\mathrm{Vx}={\left(\mathrm{Kz}/\mathrm{Kx}\right)}^{0.5}$$

##### Flight Time and Mirror Reflections

The combined effect of *Kz* (ion beam energy) and *Lz* (analyzer width) determines two crucial parameters, flight time (*T*) and number of ion reflections between ion mirrors (*N*) as described by eqs 3 and 4

3 $$T=\mathrm{Lz}/\mathrm{Vz}=\mathrm{Lz}\times {\left(m/2\mathrm{zeKz}\right)}^{0.5}$$

4 $$N=\mathrm{Lz}/\mathrm{Lx}\times \mathrm{Vx}/\mathrm{Vz}=\mathrm{Lz}/\mathrm{Lx}\times {\left(\mathrm{Kx}/\mathrm{Kz}\right)}^{0.5}$$

While flight time is mass-dependent, the trajectory itself is solely controlled by the potentials and energies per charge However, the actual number of mirror reflections is also limited by the physical dimensions of the OA, detector, and their rims.

##### Transmission of Long Ion Packets

The number of *N* reflections is controlled by the mean ion beam energy per charge *Kz* in the *Z*-direction (eq 4), once the analyzer geometry and *Kx* are chosen (*Lx* = 500 mm, *Lz* = 230 mm between OA and detector centers, and *Kx* = 6600 V). At narrow energy spread *dKz* there occur narrow “resonance” zones where ion packets successfully reach the detector. The analyzer and OA dimensions were optimized for *Kz* = 22 V, corresponding to *N* = 8 mirror reflections, where the transmitted packet length reaches 22 mm. The width of this optimal *Kz* zone is 2 V, significantly larger than the energy spread (*dKz*) within a continuous ion beam, typically maintained below 0.5 V for ion currents under 1 nA. This allows for proper tuning of *Kz* (controlled by the gas cell DC bias) to ensure transmission of 22 mm long ion packets without losses.

##### Ion Packet Focusing onto Detector (Figure 1A)

Simulated ion trajectories are shown in Figure 1A, illustrating their focusing onto the detector. This focusing is achieved by the OA lens, without using any focusing means in the analyzer. The MRT drift space lacks dedicated Z-focusing mechanisms for good reason, that is, any deflecting or focusing elements are chromatic with respect to *Kz* energy. Instead, to improve ion transmission and ensure ions bypass the detector rim effectively, a single trans-axial lens is positioned at the OA exit. Lens aberrations are compensated by a curved accelerated field. This lens focuses ion packets of length *Pz* onto the detector center. However, due to the inherent energy spread (*dKz* ≈ 0.5 V), the packets do not converge to a single point, as shown by simulated ion trajectories 12 in Figure 1A. Based on eq 5, the estimated packet width (*dZ*) on the detector is approximately 6 mm

5 dZ=Lz×dVz/Vz=Lz×dKz/2Kz

This 6 mm packet width is significantly smaller than the 31 mm trajectory Z-pitch per reflection, allowing for efficient bypass of the detector rim.

The analyzer transmission is verified against variations of the gas cell bias as illustrated in Figure 1D. The “apparatus function” of the analyzer appears approximately 2 V wide. Even though higher ion currents (1 nA) shift the energy distribution slightly (compare curves at various deeming DRE factors), the lossless ion transmission is ensured in this ion current range, so as for the wide range of molecular masses - from small molecules to heavy proteins, at least up to 150 kDa.

##### OA Duty Cycle and Conversion Efficiency

Figure 1C illustrates the efficiency of pulsed conversion in the instrument. When using continuous ion beams at the OA entrance, the geometrical duty cycle (DC) is defined as the ratio of the effective ion packet length (*Pz*) to the analyzer length (*Lz*) (eq 6). This geometrical DC directly corresponds to the pulsed conversion efficiency for the heaviest species in the spectrum at *(M*/*Z)*max, whose flight time within the MRT analyzer matches the OA pulsing period. However, for ions with lower mass, the conversion efficiency (Eff) decreases as their velocity increases, being inversely proportional to the square root of mass at a constant ion beam energy, as summarized by eq 7.

6 DC=Pz/Lz

7 $$\mathrm{Eff}=\mathrm{DC}\times {\left[\left(M/Z\right)/\left(M/Z\right)\max\right]}^{0.5}$$

At the optimal *N* of 8 reflections, the analyzer exhibits a geometrical duty cycle of 10% and an average conversion efficiency (per mass spectrum) of approximately 5%. This efficiency has been confirmed in direct current measurements on OA and detector.

#### Calibration

The multireflecting time-of-flight analyzer was externally calibrated over the acquisition mass range (50–1200 Da) before analysis with a sodium formate mixture using a multipoint calibration. Lock mass consisting of Leucine-enkephalin was delivered to the reference sprayer of the MS ESI source at a flow rate of 20 μL/min and was sampled every 60 s for 0.3 s.

### Simulations

Figure 2 presents the key results from ion optical simulations, showing that the formation of nearly parallel and narrow ion beam at the OA entrance is the dominant factor controlling the resolving power of the instrument.

**Figure 2:**
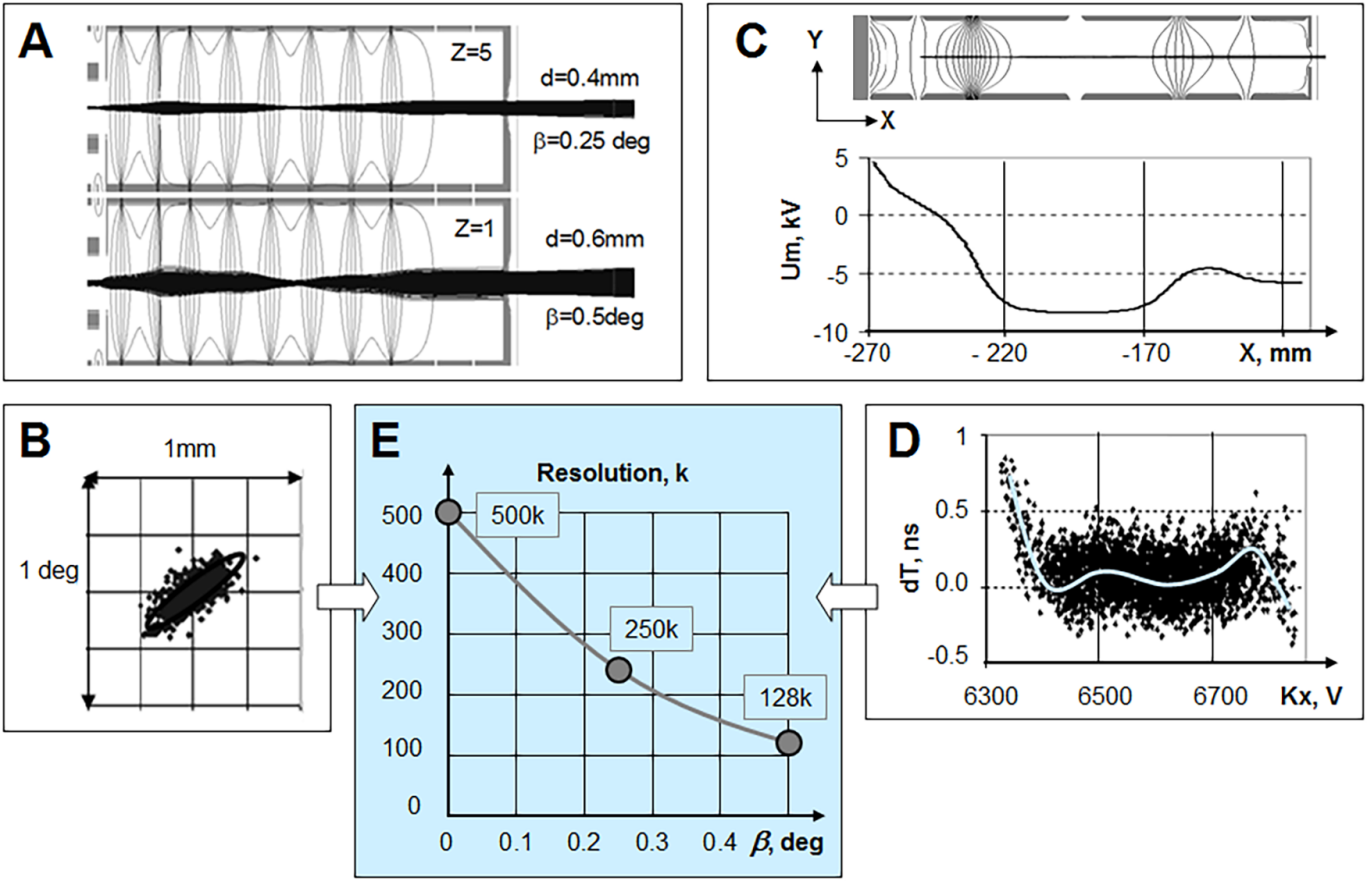
Relation between ion beam quality and analyzer resolution: (A) Simulated trajectories of ion beam at the OA entrance for *z* = 5 and *z* = 1 charged and *m*/*z* 1000 ions (vertical scale magnified). (B) Ion beam emittance in the OA for *z* = 1 ions. (C) Ion mirror fields - equipotential lines and axial potential distribution. (D) Time-energy diagram on the detector corresponding to the emittance of z = 1 ions. (E) Resolving power (*R*) vs angular divergence (β) of the continuous ion beam. Numbers in Panels (A, E) are presented at FWHM.

#### RF Compression in the Gas Cell and Ion Beam Quality

The quality of the continuous ion beam exiting the gas cell directly influences both the efficient transmission into the OA and the overall resolution. This quality is determined by the effectiveness of ion compression within a compact 3 mm diameter quadrupole utilizing radial RF fields. To maintain efficient compression at high ion currents (up to 1 nA), an axial DC field is incorporated within the cell. This counteracts charge buildup and space-charge induced ion beam expansion.^29,30^ Notably, the RF compression strengthens with increasing ion charge due to its opposition to the charge-independent thermal diffusion. The parameters of ion beams exiting the gas cell were simulated at *F* = 5 MHz, *V*_p‑p_ = 1200 V, for z = 1 and *z* = 5 ions with *m*/*z* 1000. These parameters were then used in the ion optical simulations presented in Figure 2A.

#### Ion Beam Parameters (Figure 2A,B)

The previously determined ion beam parameters from the gas cell simulation were used to model ion trajectories within the lens system and OA. The lens is optimized to focus the ion beams to parallel trajectories after forming an initial inner crossover. The initial tighter beam with *z* = 5 exhibits minimal broadening through the ion optics and fully passes the 0.9 mm collimating aperture at optics exit. At the OA center, this beam is characterized by a diameter (*d*) of 0.4 mm and noncorrelated angular divergence (β) of 0.25° (FWHM). Singly charged ions (*z* = 1) experience a lower degree of compression, resulting in a wider beam, still passing the exit aperture with a transmission of 97%. The beam parameters at the OA center for *z* = 1 are *d* = 0.6 mm and β = 0.5°. The corresponding simulated beam emittance in coordinate-angle space is depicted in Figure 2B. It is worth noting that the simulated axial energy spreads appear notably narrower (0.1 eV) than experimentally measured values (<0.3 eV). Also, all simulated distributions are close to Gaussian ones.

#### Defining Analyzer Aberrations

The electric fields within the OA and mirrors are designed to achieve both spatial confinement in the *X*–*Y* plane and time-of-flight focusing of ion packets on the detector. These fields are optimized to minimize aberration coefficients, which quantify the impact of initial ion packets spreads. As an example, time aberration coefficients include *T*|*k*, *T*|*y*, and *T*|*a*, denoting first-order effects of energy, spatial, and angular spreads, along with their cross-terms, e.g., *T*|*ky* representing the joint second-order effect of energy and spatial spreads. The order to which these aberrations are compensated directly influences the overall analyzer performance. For example, achieving *T*|*k* = *T*|*kk* = *T*|*kkk* = 0 signifies third-order energy focusing. A scenario with *T*|*k* = *T*|*y* = *T*|*a* = 0 and all second-order cross-terms (*T*|*kk*, *T*|*yy*, etc.) also equal to zero would represent full second-order focusing, encompassing correction for these energy-spatial spread interactions. In essence, a more sophisticated mirror design characterized by minimized aberration coefficients translates to superior ion packet focusing and an enhanced combination of analyzer resolution and sensitivity.

#### MRT Quality

To achieve exceptional performance, the analyzer utilizes a design that incorporates full third-order focusing and fourth-order energy focusing for the mirrors.^31,32^ The OA, on the other hand, is specifically designed to minimize the angular divergence of ion packets to within 1 mrad at an initial beam diameter of 1 mm. Some key features of ion mirror fields are illustrated in Figure 2C.

#### Entrance/Exit Lenses

Referring to Figure 2C, both the gridless OA and mirrors inherently possess exit lenses due to the field transitions between the elements and the field-free drift spaces. These lenses are intentionally designed to be stronger than those that would occur with a smooth field decay, as witnessed by axial potential distributions in Figure 2C. The stronger OA lens focuses the ejected ion packets, ensuring parallel ion trajectories with <1 mrad angular divergence. The stronger mirror lens focuses ions spatially at the reflection plane. Additionally, a deeper, attractive lens potential compared to the drift region proves beneficial for achieving fourth-order time-per-energy focusing, assisted with the so-called immersion lens and a fine structure of reflecting field.^32^ The combination of two lenses allows to reduce the maximal used potential under 8 kV at −6 kV acceleration voltage aiding the analyzer stability against breakdown.

However, all lenses inherently introduce a T|yy aberration due to the trajectory bending and associated changes in axial velocity. Fortunately, the curvature of the reflecting and accelerating fields within the OA and mirrors effectively compensates for this lens aberration, as shown in Figure 2C.

#### Simulating Time Spread in the Analyzer

Figure 2D shows the ion flight time vs ion energy on the detector. The plot is obtained in combined ion optical simulations encompassing both the analyzer and the OA, while using the ion beam parameters obtained in simulation of continuous ion beam past the RF gas cell at *z* = 1 and *m*/*z* 1000, shown in panel 2B. The time spread primarily originates from the ion beam angular divergence, while the energy spread and a slightly waved average T-K curve originate from the spatial spread of the ion beam. Despite the visual time spread spanning for 1.2 ns, the flight time distribution corresponds to FWHM <0.5 ns at 125 μs average flight time, theoretically predicting *R* ∼ 130,000 resolving power for singly charged ions at *m*/*z* 1000. Note that simulations do not account for the detector time spread and pulse jitters.

#### Turn-Around Time Limit

Similar simulations were made for a range of Gaussian angular distributions at variable FWHM (β). The spatial beam distribution was preserved at FWHM (*d*) ∼ 0.6 mm level, matching one for *z* = 1 ions in previous simulations. The analyzers aberration limit was simulated as *R* = 500,000 at zero angular divergence. The plot of Figure 2E illustrates that the analyzer resolution is primarily controlled by the ion beam angular divergence, in turn controlling the turnaround time of ion packets. Tighter beam compression in RF ion guide allows to form a lower divergence ion beam (Figure 2A), improving the analyzer resolution.

## Results

### Resolution

Figure 3A shows the MS/MS spectrum of [Glu^1^]-Fibrinogen peptide B (GFP), (EGVNDNEEGFFSAR, C_66_H_95_N_19_O_26_, Sigma-Aldrich, St. Louis, MO) infused at a concentration of 10^–7^ M. The doubly charged precursor was selected by the AQ at 5 Th mass window to admit the entire isotopic envelope. Precursor ions were injected into the CID collision cell at 27 V energy per charge. The product spectrum primarily contains singly charged fragments, dominating y-series. The maximal intensity of fragments corresponds to λ ∼ 1 ion/peak/shot, with the vast majority of fragment peaks corresponding to λ ≪ 1. This range of l corresponds to undisputedly correct application of the centroiding method.

**Figure 3:**
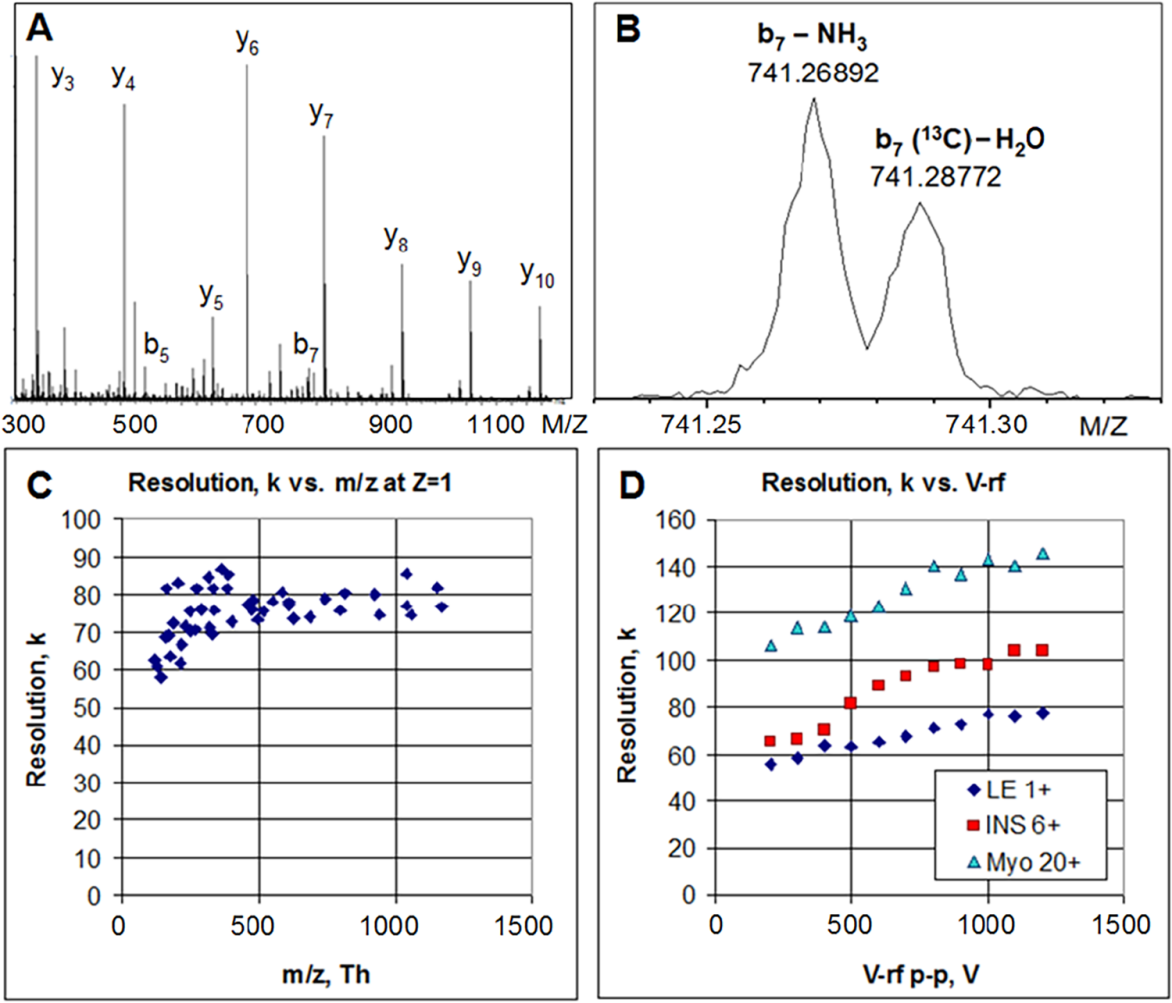
Resolution of Xevo MRT mass spectrum. (A) MS/MS spectrum of [Glu^1^]-Fibrinogen peptide B in a wide mass range. (B) Detail spectrum of *m*/*z* 741.2 (from A) exhibiting ∼8% intensity of the base peak showing fine isobars, the data comprises a histogram of ∼6900 individual Tof transients corresponding to a total acquisition time of 1 s. (C) Resolution for singly charged fragments vs their *m*/*z*. (D) Resolution vs RF amplitude (peak–peak) for ions of various charge states of Leucine Enkephalin LE (*z* = 1), Insulin INS (*z* = 6) and Myoglobin Myo (*z* = 20).

Figure 3B shows a 0.1 Th wide region of the MS/MS spectrum to illustrate the separation of close isobaric species, with the b7-NH_3_ and to the first ^13^C isotope of b7-H_2_O fragments at *m*/*z* 741.269 and 741.288 Th, spaced 19 mTh apart. The intensity of this ion is ∼8% of the base peak intensity of the GFP spectrum. The data comprises a histogram of ∼6900 individual Tof transients corresponding to a total acquisition time of 1 s. Figure 3C illustrates the resolution of the singly charged fragment ions, which stayed constant around 80K over the complete mass range and dropped marginally at lower *m*/*z* end (corresponding to shorter flight times) due to the effect of residual jitters and time spread at 4 GS/s digitization estimated at a 0.15 to 0.2 ns level. The effect is less pronounced compared to benchtop singly reflecting TOF systems because of the longer flight time in o-MRT (125 μs at *m*/*z* = 1000).

In Figure 3D, the effect of charge state and RF amplitude in the gas cell on resolution are shown for selected peptides and proteins. Separate infusions of each analyte were completed and mass spectra were acquired over 60 s at 1 Hz MS mode of acquisition for each collision cell RF (V) with the ion beam attenuated to ∼0.1 ions per push to ensure accurate measurement of mass resolution. Mass spectra of monitored charge states for each peptide/protein at 1200 V RF are shown in Supporting Information (Figure S1). Higher RF amplitudes improve the ion beam compression in the CID cell and the compression improves for higher charge states.^29,30^ The resolution reaches a maximum of *R* = 140 k at *z* = 20, at V-rf = 1200 V p-p at 5 MHz frequency for myoglobin. The effect is explained by a tighter compression of ion beam in the RFG, which reduces the ion beam divergence in the OA, as illustrated in Figure 3D. Simulations presented in Figure 2A explain resolution improvement at smaller ion beam divergences.

### Sensitivity, Dynamic Range, Mass Accuracy, and MS/MS Resolution

The post-CID ion optics and the gridless analyzer are characterized by lossless ion transmission. Ion transmission was confirmed via direct current measurements on the CID cell and OA electrodes. Ion current proportionality was maintained up to a value of 1 nA. Despite the moderate duty cycle of 10% of the OA, the instrument provides notably higher sensitivity compared to TOF analysers using grids. Detector improvements have allowed the handling of very high ion fluxes, approaching one billion ions/s. As a result, the instrument extends the dynamic range of the analysis, which is beneficial for the analysis of complex samples.

LC-MS sensitivity, dynamic range and mass accuracy were demonstrated using a NIST 1950 SRM plasma extract spiked with deuterated internal lipid standards (EquiSPLASH LIPIDOMIX, Avanti Research, Alabaster, AL). The lipids were separated using Reversed Phase chromatography over a 6 min gradient with the column outlet connected directly to the Xevo MRT mass spectrometer. The LC flow rate was 400 μL/min and injection volume 5 μL. The LC-MS parameters are provided in Supporting Tables S1 and S2. LC-MS data were acquired using a Data Independent Acquisition (DIA) strategy, MSE.^33^ Wide mass range data were acquired into separate low and high collisional energy data streams. Fragment and parent ions correlate in retention time *t*_R_, providing additional confirmation for compound identification. Data from representative EquiSPLASH deuterated components, phosphocholine (15:0–18:1­(d7) PC; PC) and lysophosphocholine (18:1­(d7) LPC; LPC) are provided in Figure 4 over the concentration range of 0.1 to 10,000 ng/mL, corresponding to sample loads from 0.2 fg to 20 pg. Exemplar spectra for the PC and LPC are provided for the lower and upper concentrations as Supporting Figures S2 and S3.

**Figure 4:**
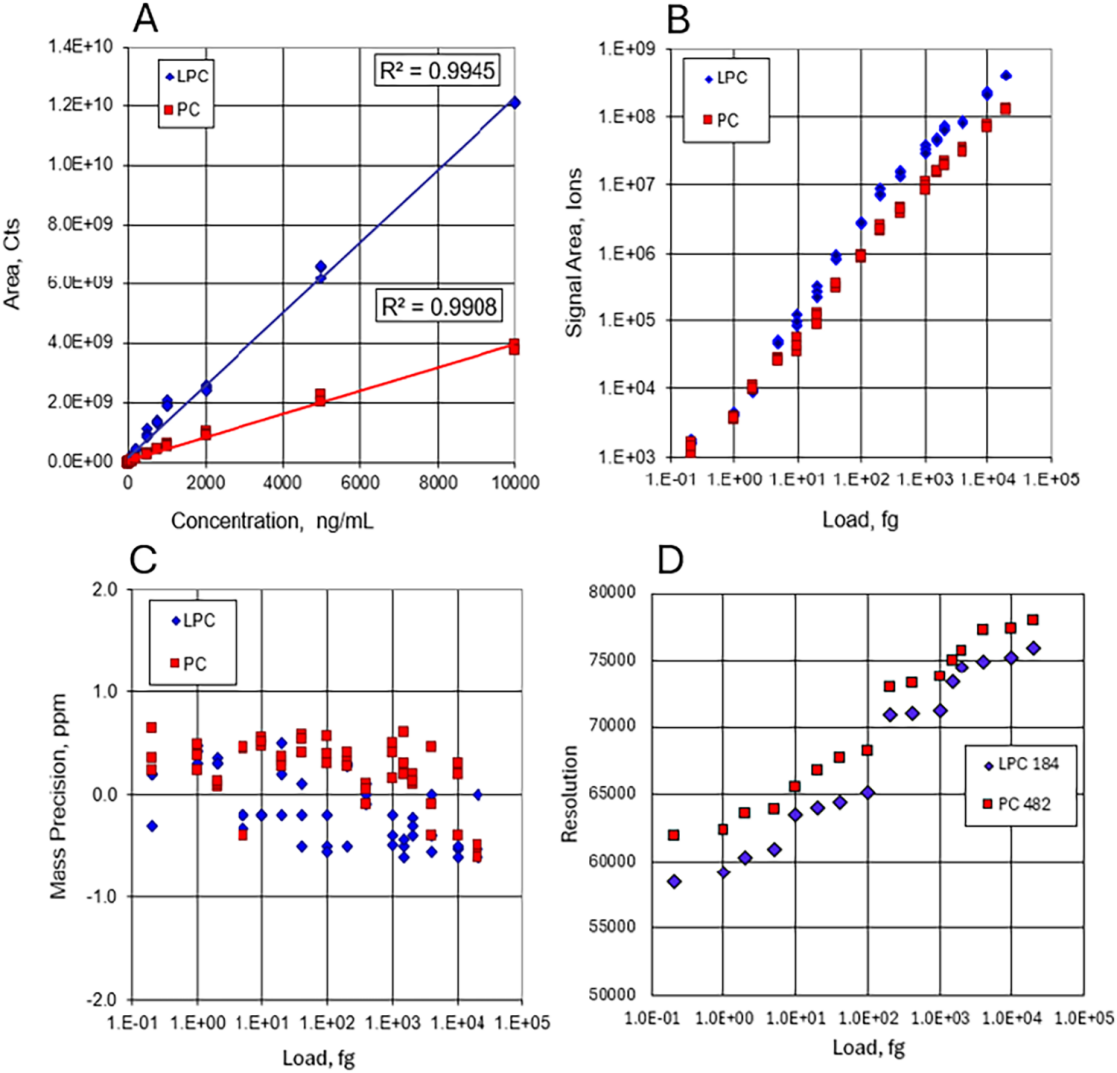
Dynamic range, mass accuracy and resolution in LC-MS analyses: (A) Linear dynamic range of the EquiSPLASH lipid components LPC and PC spiked in NIST plasma. (B) Analyte signal area dynamic range for LPC and PC. (C) Mass accuracy vs sample load for LPC and PC. (D) Mass resolution vs sample load for MS/MS data from LPC *m*/*z* 184 and PC *m*/*z* 482 fragment ions.

Both compounds demonstrate a linear dynamic range of 5-orders with *R* ^2^ > 0.99 for both compounds (Figure 4A). Sensitivity at the lowest level measured is also presented as signal area (Figure 4B). At lower concentrations, the chemical noise increases and thus impacts on SNR. However, the high resolution afforded by the Xevo MRT provides a means of enabling sufficient combination of sensitivity (1000 ions detected in LC peak) and spectral selectivity against background species, further enhanced by *t*_R_-correlated detection of fragments. This is highlighted at the lowest level measured (0.1 ng/mL) for the LPC and PC standards. The mass accuracy for both compounds was also determined over the concentration range, with the mass error consistently being returned at <1 ppm, shown in Figure 4C. The mass resolution was a FWHM measurement taken from the MS/MS data of the LPC 184 *m*/*z* and PC 482 *m*/*z* fragments. As can be seen in Figure 4D, most measurements achieved *R* ∼ 60,000–80,000 and only fell to 58,413 for the lowest 0.2 fg LPC loading.

### Note on the Maximal Dynamic Range of o-MRT

The demonstrated DR = 10^5^ is the application-specific dynamic range corresponding to realistic concentrations of lipids in plasma. A much higher dynamic range (above 8 orders of magnitude) has been experimentally proven in case of injecting pure standards and without plasma matrix. In this non presented experiments, the upper loads of injected standards were increased by 100-fold to 1 mg/mL (diluted by ∼10-fold after LC), while detector saturation was prevented with a time-alternated suppression in the interface (DRE method). The lowest detectable concentrations corresponded to 10 pg/mL, yielding signals with the total intensity of 100 ions forming a smooth profile of selected ion current.

### Application to LC-MS Lipidomics Analysis

Lipids are an integral part of human biology and represent around a third of all known metabolites.^34^ Strongly influenced by endogenous and/or exogenous factors, they are highly regulated through a variety of complex biological processes, including energy storage, cellular signaling and cell–cell interactions.^26^ Lipids exhibit themselves in a variety of classes, e.g., fatty acyls, glycerophospholipids, sphingolipids, and occur over a wide dynamic range of 10^6^ or more from nanomolar to attomolar levels.^35^

To demonstrate the applicability of the Xevo MRT for lipidomic analyses, the same plasma lipid extract spiked with varying concentrations of deuterated lipid standards was used here as in the experiment detailed above. The LC-MS data were acquired, peak-picked and database searched accounting for endogenous and the deuterated internal standard components, using waters_connect (Waters Corporation, Wilmslow, U.K.) and Progenesis QI (Waters Corporation, Wilmslow, U.K.) software packages. A representative Total Ion Chromatogram (TIC) is shown in Figure 5A, with eXtracted Ion Chromatograms (XIC) for the 15:0–18:1-d7–15:0 TAG precursor with *m*/*z* 829.7 at 10,000 and 0.1 ng/mL provided, shown in Figure 5B,C, respectively, providing a SNR of 10 at 0.1 ng/mL. Representative spectra of the TAG precursor at 10,000 and 0.1 ng/mL are provided as Supporting Figure S4.

**Figure 5:**
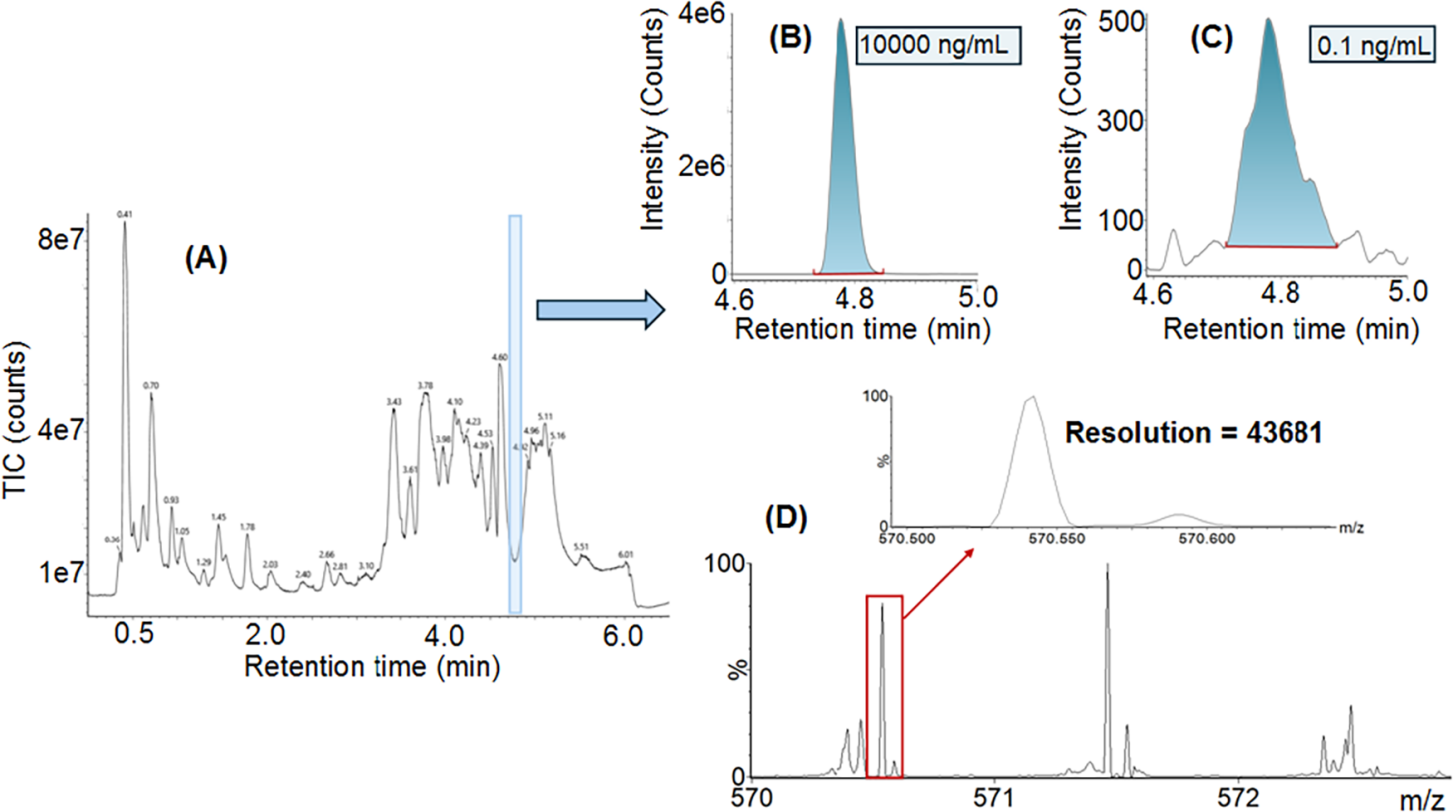
Representative lipidomic example based on the NIST SRM 1950 plasma spiked with EquiSPLASH, showing TIC (A) along with XIC’s of the EquiSPLASH 15:0–18:1­(d7)-15:0 TAG at 10,000 (B) and 0.1 ng/mL (C). The fragment ion spectrum for the lowest studied 0.1 ng/mL concentration is shown (D), along with its associated resolution at FWHM in zoom. LC-MS experimental details are provided as Supporting Tables S1 and S2.

Structural elucidation of compounds based on fragment ion data relies strongly on unadulterated or pure peak detections. This is demonstrated with an example *m*/*z* 570.54 fragment ion peak corresponding to the TAG internal standard, which is resolved from interfering peaks which could be attributed to coeluting compounds and chemical background as demonstrated in Figure 5D. Even at this lowest analyte level, the fragment ion showed a resolution of 43,681, partially affected by close isobars of the coeluting background. Note that in the absence of mass interferences, the resolution is not affected for small intensity peaks as presented in Figure 3D

Additional identification confidence is provided with the fragment ions being confirmed with a mass tolerance of ±2 ppm following database searching. This level of mass accuracy allows for a significant reduction in false positive identifications. The mass error distributions shown Supporting Figure S5 result in 54% and 42% of the fragment ions being <1 ppm for the 10,000 ng/mL and 0.1 ng/mL levels, respectively.

Identification scores resulting from database searching show only a marginal change over the concentration range, as illustrated by the results shown in Supporting Figure S6. The identification score is a combination of corresponding fragment ions in addition to mass accuracy (precursor and fragment ions) and isotopic fit. At the lowest concentration of 0.1 ng/mL, where chemical noise becomes more apparent, the combination of high mass resolution and mass accuracy increasingly assists with confident compound identification.

Example precursor and product ion spectra of the midlevel concentration spikes from two of the deuterated lipid standards, 18:1-d7 Lyso PC and C15 Ceramide-d7 (d18:1-d7/15:0), respectively, are provided in Supporting Figures S7 and S8.

## Discussion and Conclusions

This paper details the fundamental design of the novel Xevo MRT mass spectrometer, highlighting its key performance characteristics and demonstrating its application in a lipidomics workflow. The Xevo MRT embodies a strategic design philosophy, effectively balancing high resolution, sensitivity, and dynamic range with practical considerations such as compact size, affordability, and operational efficiency.

A deliberate choice was made to prioritize a resolution *R* of 100,000 compared to other MRT designs, both commercial and prototypic. This, coupled with the inherent space-saving nature of MRT flight paths, allows for a significantly reduced footprint (in particularly, fitting standard bench depth of 80 cm), especially when contrasted with traditional single-reflection TOF instruments that can extend up to 3 m to reach *R* = 80,000.^36^ This resolution is achieved within a relatively short 4-m flight path through the integration of two key innovations: strong RF compression and centroid data acquisition.

While the pulsed conversion method yields a moderate conversion efficiency of up to 10%, it offers substantial advantages in operational speed, charge throughput, and total dynamic range (TDR). Notably, it circumvents the limitations of trap converters, namely slow pulsing frequencies of typically 200 Hz and low charge capacity of around 50,000 charges.^16^ This advantage is critical for analyzing complex mixtures where high throughput and TDR are paramount.

Efficient ion beam compression via strong RF within the gas cell results in exceptionally short turnaround times post-OA,^29,30^ enabling the formation of sharp ion packets with time spreads as low as 0.2 ns.^24^ The centroid format used for ADC data acquisition effectively minimizes the detector’s time spread,^24^ allowing for the accurate recording of these fast ion packets with minimal additional broadening (estimated <0.1 ns).

The instrument system also achieves high sensitivity through enhancements in the ion source and interface, particularly when coupled with nano-LC and nano-ESI tips, readily reaching 3 × 10^8^ ions/ng at 0.5 μL/min. This translates to strong ion currents (approaching 1 nA) within the gas cell, resulting in detected ion fluxes near 1 × 10^9^ ions/s.

To fully exploit this wide dynamic range, the system incorporates a long-life detector and a high dynamic range data acquisition system employing a dual-gain amplifier.^37^ This configuration enables the recording of signals with λ values up to 5000 as summarized in Figure 4, accounting for an 8 kHz pulsing rate, achieving an in-spectra dynamic range of 10^6^ and a TDR of 1 × 10^8^ in one-second summed spectra. Remarkably, subppm mass accuracy is maintained across this entire dynamic range. The exceptional TDR of the compact-MRT system compares favorably with other instruments,^13^ providing a significant benefit for the analysis of complex samples such as encountered in metabolomics, lipidomics, and proteomics applications.

While the resolution is lower than that of an experimental o-MRT prototype,^24^ it is achieved with accelerated transit times in the collision cell, facilitating faster MS/MS analyses. Although the resolution value may appear modest compared to FTMS instruments, it is crucial to emphasize that it is attained with a short 4-m flight path, a rapid flight time of 125 μs at *m*/*z* 1000 Th, and a high repetition rate of 8 kHz, representing substantially increased speed and charge throughput. The short flight path is particularly advantageous for reducing ion scattering when analyzing large proteins, a key application in mass spectrometry.^24^ The fast repetition rate is also highly beneficial for achieving high throughput and dynamic range in analyses.

Biological samples present inherent analytical challenges due to wide dynamic ranges, diverse compound types, matrix effects, and varying analyte properties. These factors impact both dynamic range and sensitivity, underscoring the necessity for mass spectrometers with substantial dynamic range capabilities for accurate measurements.

The identification and quantification of lipids in biological samples are particularly demanding.^38,39^ The complexity of these samples, compounded by wide dynamic range and matrix effects, often leads to chromatographic and mass separation overlaps, and instrument sensitivity for lipids can thus vary significantly. These factors highlight the critical need for mass spectrometers with high dynamic range, resolution, and mass accuracy for reliable lipid analysis, as effectively demonstrated by the instrument’s performance in a complex plasma lipid sample shown in Figure 5.

## Supplementary Material



## Notes

LC parameters used for acquiring the NIST SRM 1950 plasma spiked with EquiSPLASH (Table S1); MS parameters used for acquiring the NIST SRM 1950 plasma spiked with EquiSPLASH (Table S2); spectra of Leucine Enkephalin (*z* = 1), Bovine Insulin (*z* = 6) and Myoglobin (*z* = 20) (Figure S1); representative MS1 spectra (continuum) for the 18:1­(d7) LPC at 10,000 ng/mL and 0.1 ng/mL concentrations (Figure S2); representative MS1 spectra (continuum) for the 15:0–18:1­(d7) PC at 10,000 ng/mL and 0.1 ng/mL concentrations (Figure S3); representative MS1 spectra (continuum) for the 15:0–18:1-d7–15:0 TAG at 10,000 ng/mL and 0.1 ng/mL concentrations (Figure S4); fragment ion mass error distributions relating to EquiSPLASH TAG for the 0.1 and 10,000 ng/mL spike concentration levels (Figure S5); identification scores returned following database searching with Progenesis QI software (Waters Corporation, Wilmslow, U.K.) over the concentration range 0.1–10,000 ng/mL (Figure S6); data Independent Analysis (DIA) MSE precursor (top) and product (bottom) ion spectra of 18:1-d7 Lyso PC at 750 ng/mL eluting at 0.53 min (Figure S7); data Independent Analysis (DIA) MSE precursor (top) and product (bottom) ion spectra of sodiated C15 Ceramide-d7 (d18:1-d7/15:0) at 750 ng/mL eluting at 2.23 min (Figure S8) (PDF)
