# Supplementary material for: A Novel Compact Multi-Reflecting Time-of-Flight Mass Spectrometer
Source: J Am Soc Mass Spectrom. 2026 Jan 26;37(3):601–11. doi: 10.1021/jasms.5c00321 (PMC12964538; doi:10.1021/jasms.5c00321)
Supplement: Supplementary file 1 [file js5c00321_si_001.pdf]

A Novel Compact Multi-Reflecting Time-of-Flight Mass Spectrometer

Anatoly N. Verenchikov [1,*], Jason Wildgoose[2], Sergey N. Kirillov[1], Aleksey V. Vorobyev[1], Vasily V. Makarov[1], Lee A. Gethings, Robert P. Tonge[2], Matthew E. Daly[2], William J. Johnson[2], James I. Langridge[2]

* anatoly.verenchikov@gmail.com

1. Mass Spectrometry Consulting Ltd, A5 JNA blv, Bar, 85000, Montenegro
2. Waters Corporation, Altrincham Road, Wilmslow, SK9 4AX, United Kingdom

Table S1. LC parameters used for acquiring the NIST SRM 1950 plasma spiked with EquiSPLASH.

LC configuration

LC System	ACQUITY™ Premier UPLC™ (flow through needle (FTN) configuration)
Analytical Column	ACQUITY Premier CSH™ C18 2.1 x 50 mm, 1.7 µm
Mobile Phase A	60:40 (ACN:Water), 10 mm ammonium formate, 0.1% formic acid
Mobile Phase B	90:10 (IPA:ACN), 10 mm ammonium formate, 0.1% formic acid
Column Temperature (°C)	55
Sample Temperature (°C)	8
Injection Volume (µL)	0.5

LC gradient

Time (min)	Flow Rate (mL/min)	%A	%B	Curve
Initial	0.4	50	50	Initial
0.25	0.4	47	53	6
2.0	0.4	45	55	6
3.5	0.4	35	65	6
3.7	0.4	20	80	1
5.0	0.4	1	99	6
5.5	0.4	1	99	1
6.5	0.4	50	50	1

Table S2. MS parameters used for acquiring the NIST SRM 1950 plasma spiked with EquiSPLASH.

Polarity	Positive (ESI)
Data Acquisition Method	MS^E (DIA)
Scan Rate (Hz)	20
Mass Range (m/z)	50-1200
Collision Energy Ramp (eV)	25-55
Capillary Voltage (kV)	1.2
Sampling Cone (V)	40
Source Temperature (°C)	120
Desolvation Temperature (°C)	500
Cone Gas (L/hr)	50
Desolvation Gas Flow (L/hr)	750
Quadrupole Profile	Auto
Lockspray Settings	Leucine enkephalin (*m/z* 556.27658) acquired as a dual point lockmass (*m/z* 556.27658 and 120.08078) every 60 s
Lockspray Collision Energy (eV)	30

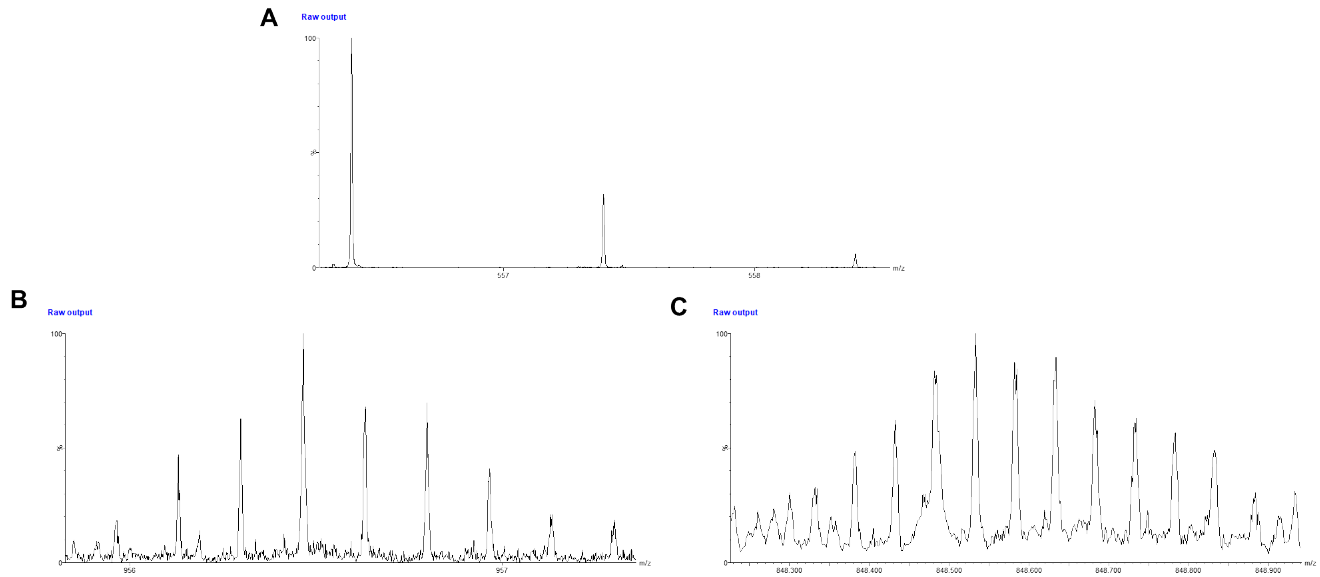

Figure S1. Spectra of Leucine Enkephalin (z = 1, A), Bovine Insulin (z = 6, B) and Myoglobin (z = 20, C). All spectra are summed over 60 seconds of 1Hz MS acquisition.

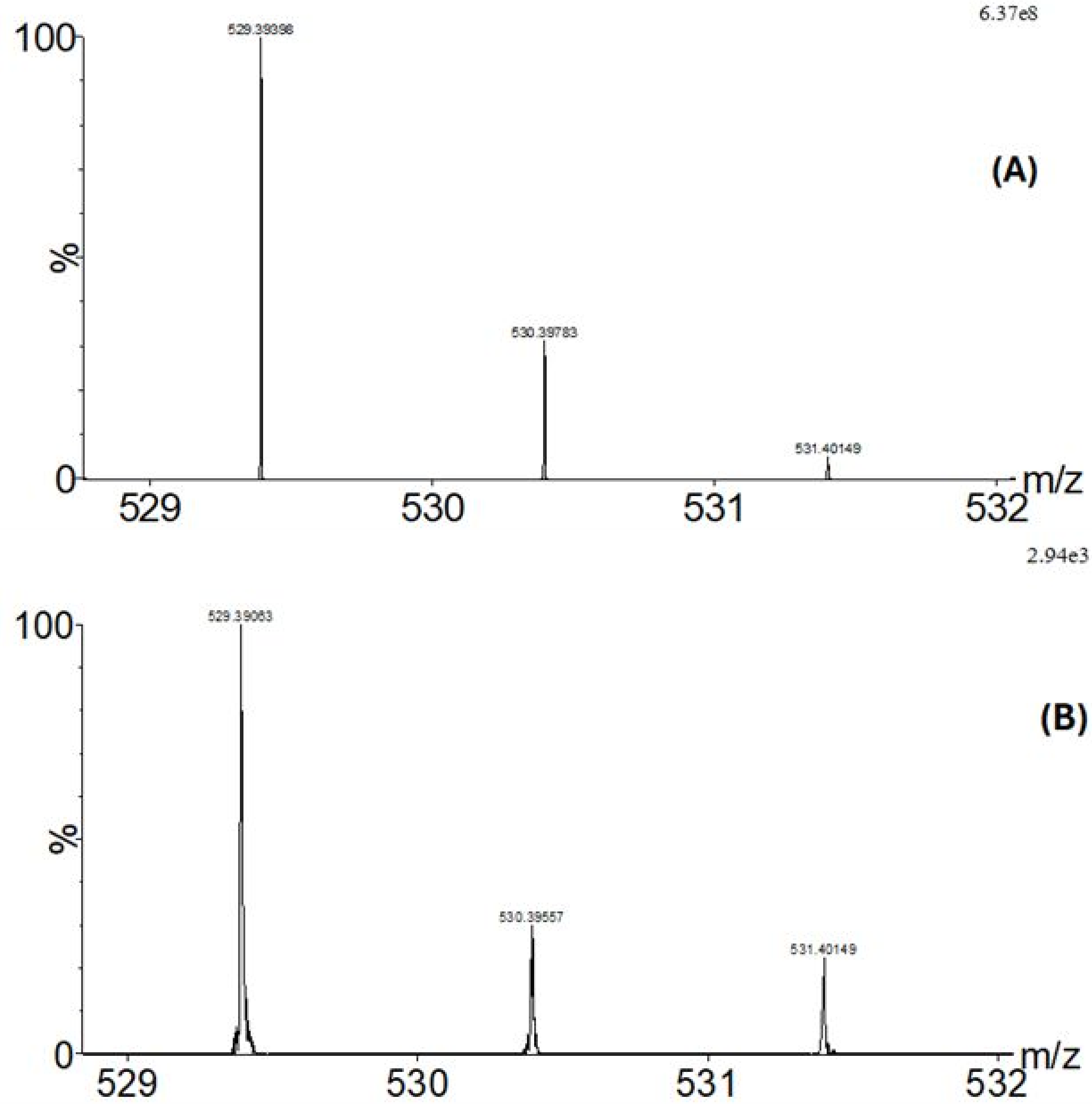

Figure S2. Representative MS1 spectra (continuum) for the 18:1(d7) LPC at 10000 ng/mL (A) and 0.1 ng/mL (B) concentrations.

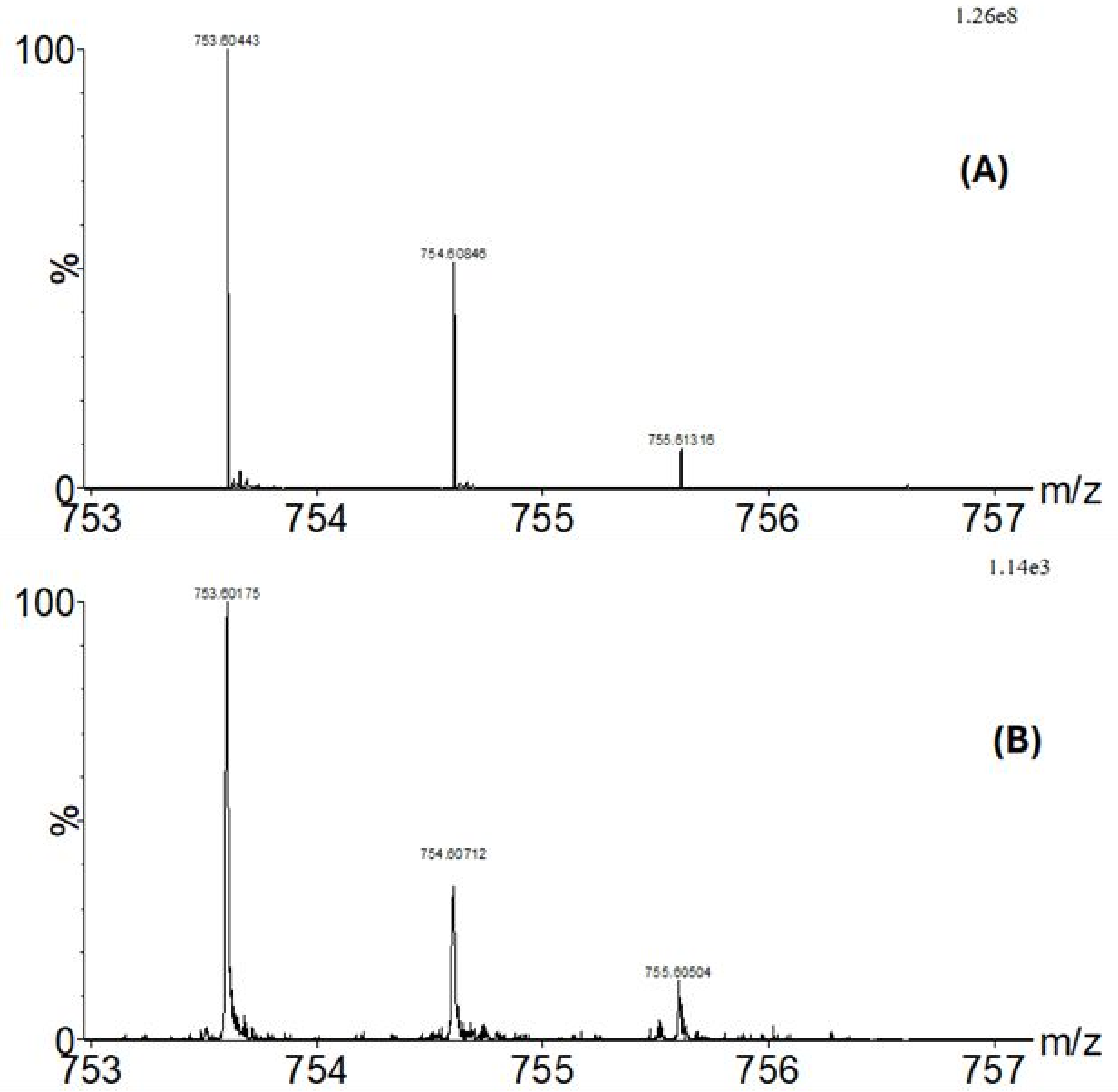

Figure S3. Representative MS1 spectra (continuum) for the 15:0-18:1(d7) PC at 10000 ng/mL (A) and 0.1 ng/mL (B) concentrations.

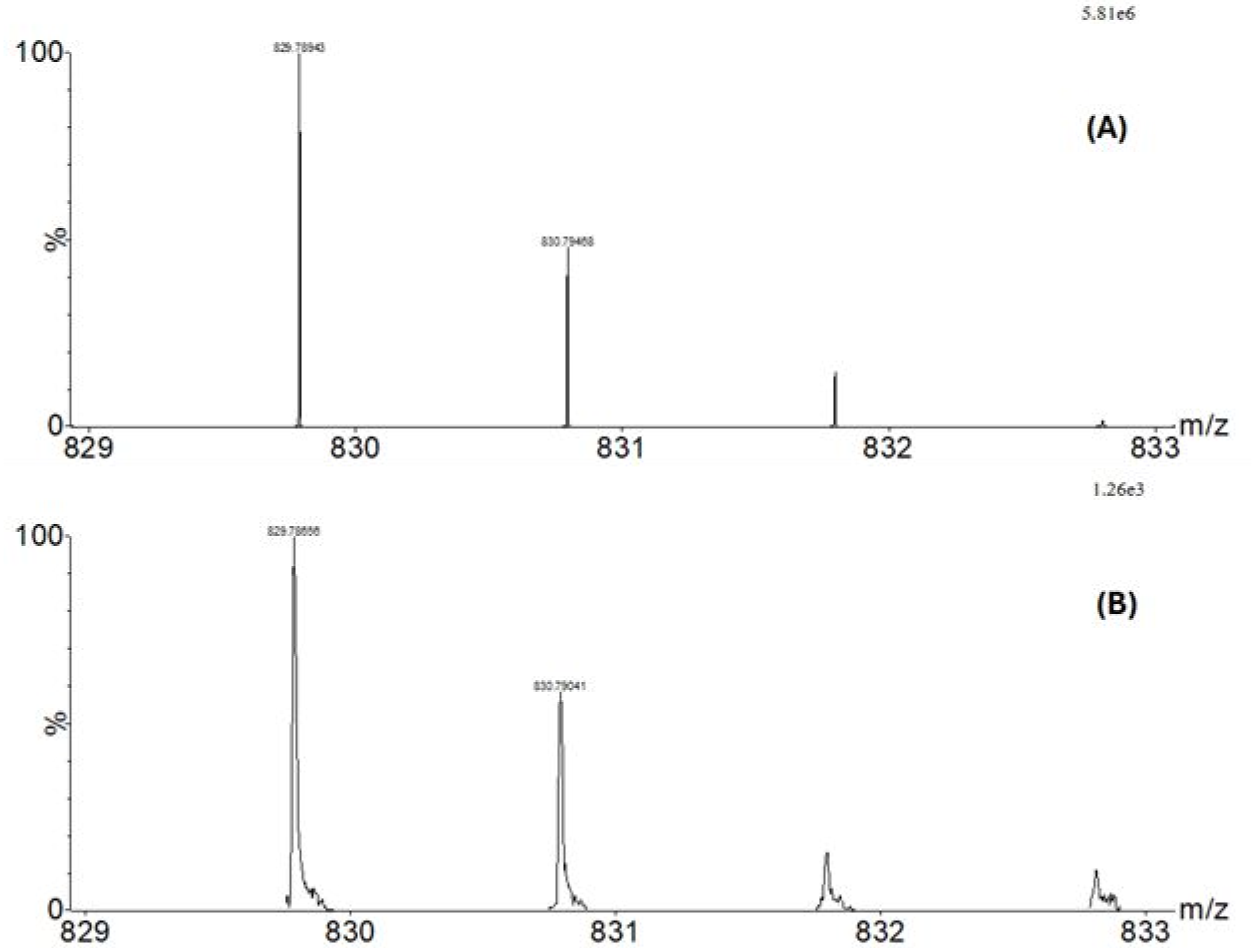

Figure S4. Representative MS1 spectra (continuum) for the 15:0-18:1-d7-15:0 TAG at 10000 ng/mL (A) and 0.1 ng/mL (B) concentrations.

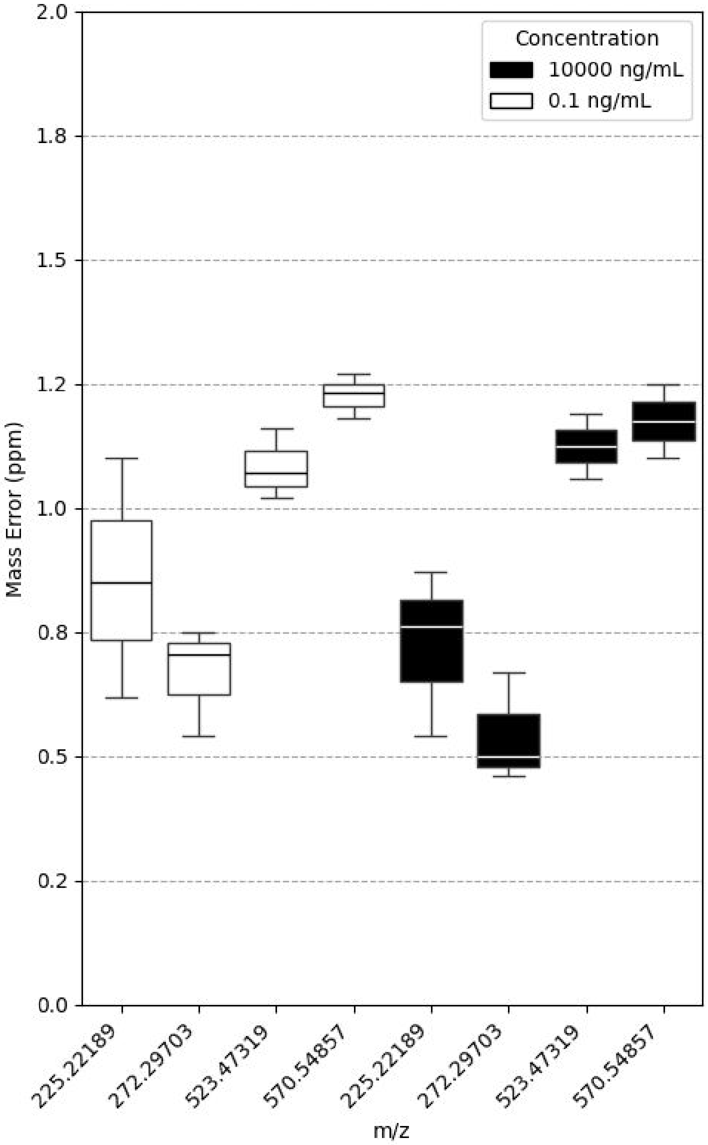

Figure S5. Fragment ion mass error distributions relating to EquiSPLASH 15:0-18:1-d7-15:0 TAG. Data comprised of all technical replicates (n = 3) for the 0.1 (white) and 10,000 ng/mL (black) spike concentration levels.

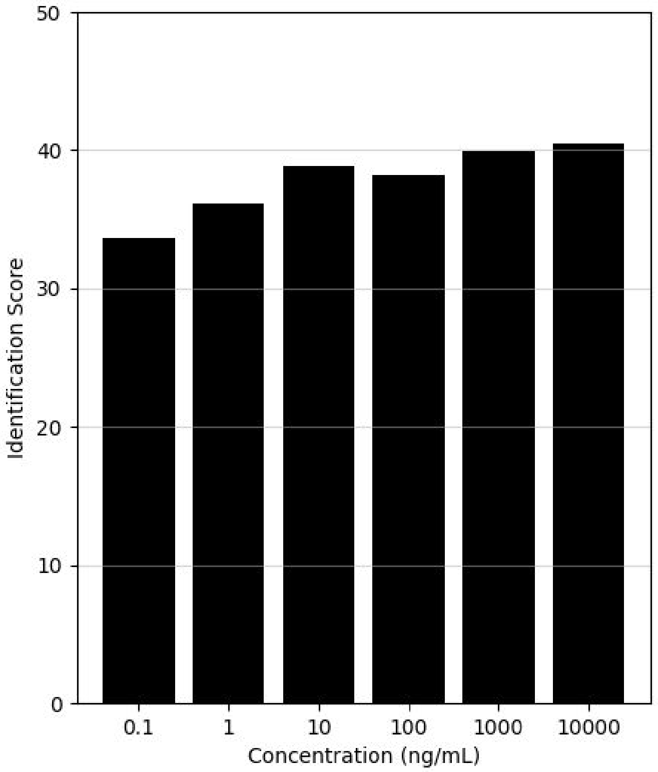

Figure S6. Identification scores returned following database searching with Progenesis QI software (Waters Corporation, Wilmslow, UK) for 15:0-18:1-d7-15:0 TAG over the concentration range 0.1 - 10,000 ng/mL using LIPID MAPS® Structure Database (https://www.lipidmaps.org/databases/lmsd), and 1 and 2 ppm precursor and product ion search tolerances, respectively.

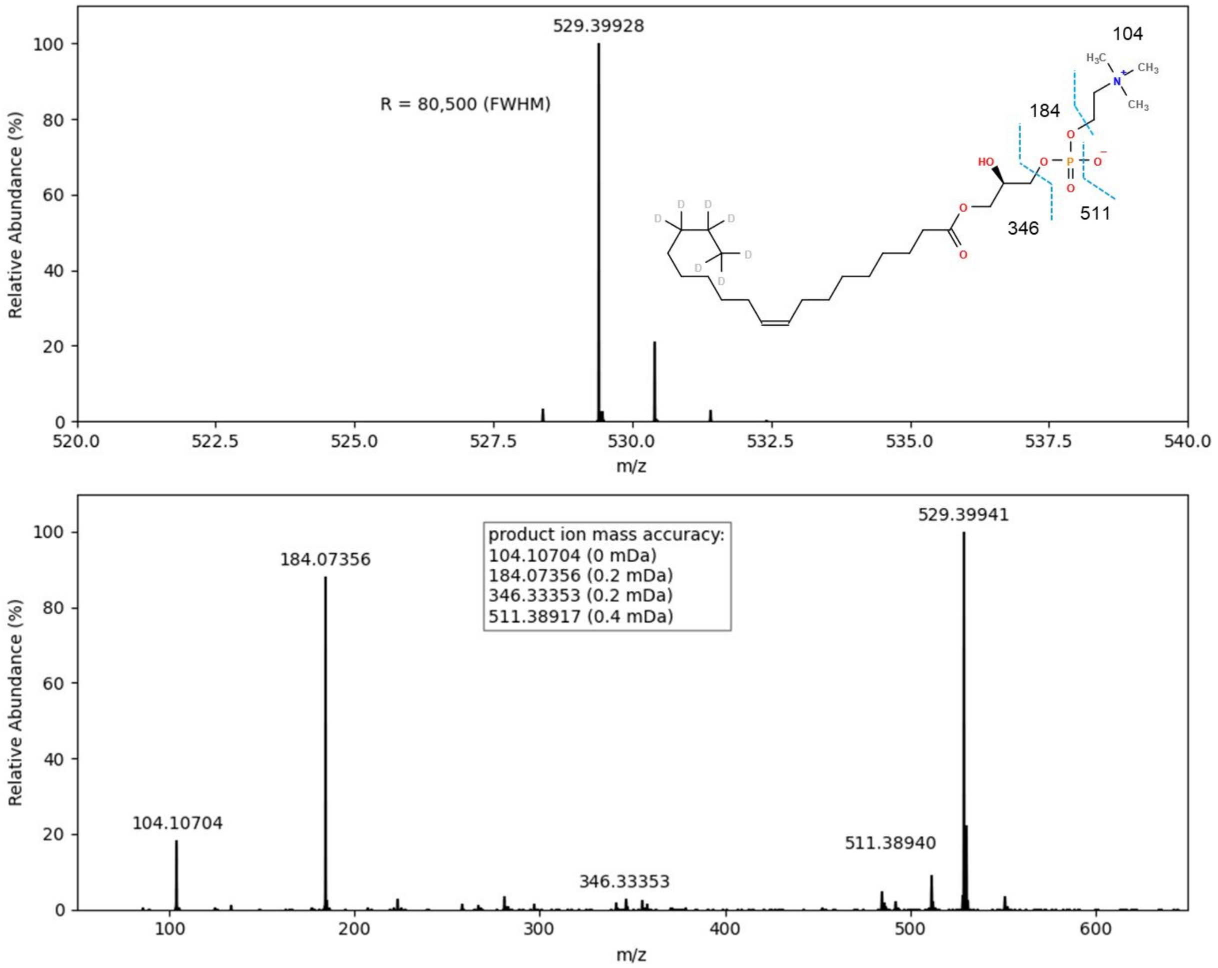

Figure S7. Data Independent Analysis (DIA) MSE precursor (top) and product (bottom) ion spectra of 18:1-d7 Lyso PC at 750 ng/mL eluting at 0.53 min. Shown inset are the chemical structure with possible collision-induced bond fragmentation patterns and product ion mass accuracy for selected product ions, respectively.

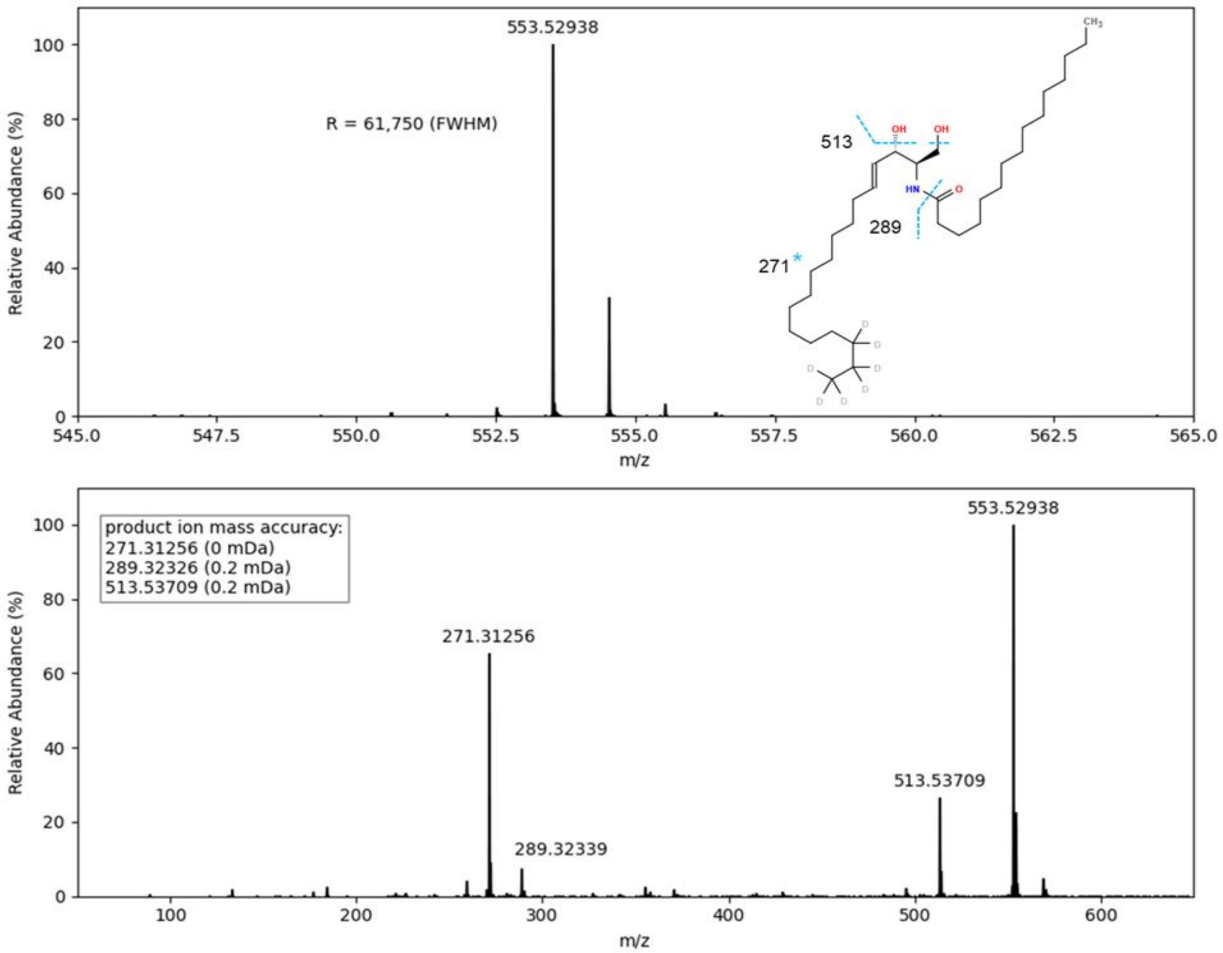

Figure S8. Data Independent Analysis (DIA) MSE precursor (top) and product (bottom) ion spectra of sodiated C15 Ceramide-d7 (d18:1-d7/15:0) at 750 ng/mL eluting at 2.23 min. Shown inset are the chemical structure with possible collision-induced bond fragmentation patterns and product ion mass accuracy for selected product ions, respectively. * Fragment 271 is produced when all three highlighted bonds dissociate.
